# An automatic pipeline for PET/MRI attenuation correction validation in the brain

**DOI:** 10.1186/s40658-023-00590-3

**Published:** 2023-11-14

**Authors:** Mahdjoub Hamdi, Chunwei Ying, Hongyu An, Richard Laforest

**Affiliations:** 1 Mallinckrodt Institute of Radiology, Washington University in St. Louis, St. Louis, MO USA; 2 Neurology, Washington University in St. Louis, St. Louis, MO USA; 3 Biomedical Engineering, Washington University in St. Louis, St. Louis, MO USA; 4 Electrical and Systems Engineering, Washington University in St. Louis, St. Louis, MO USA

**Keywords:** Quantitative brain PET, PET attenuation correction, PET/MRI, PET/CT, FreeSurfer brain atlas, Virtual synthetic PET imaging

## Abstract

**Purpose:**

Challenges in PET/MRI quantitative accuracy for neurological uses arise from PET attenuation correction accuracy. We proposed and evaluated an automatic pipeline to assess the quantitative accuracy of four MRI-derived PET AC methods using analytically simulated PET brain lesions and ROIs as ground truth for PET activity.

**Methods:**

Our proposed pipeline, integrating a synthetic lesion insertion tool and the FreeSurfer neuroimaging framework, inserts simulated spherical and brain ROIs into PET projection space, reconstructing them via four PET MRAC techniques. Utilizing an 11-patient brain PET dataset, we compared the quantitative accuracy of four MRACs (DIXON, DIXONbone, UTE AC, and DL-DIXON) against the gold standard PET CTAC, evaluating MRAC to CTAC activity bias in spherical lesions and brain ROIs with and without background activity against original (lesion free) PET reconstructed images.

**Results:**

The proposed pipeline yielded accurate results for spherical lesions and brain ROIs, adhering to the MRAC to CTAC pattern of original brain PET images. Among the MRAC methods, DIXON AC exhibited the highest bias, followed by UTE, DIXONBone, and DL-DIXON showing the least. DIXON, DIXONbone, UTE, and DL-DIXON showed MRAC to CTAC biases of − 5.41%, − 1.85%, − 2.74%, and 0.08% respectively for ROIs inserted in background activity; − 7.02%, − 2.46%, − 3.56%, and − 0.05% for lesion ROIs without background; and − 6.82%, − 2.08%, − 2.29%, and 0.22% for the original brain PET images’ 16 FreeSurfer brain ROIs.

**Conclusion:**

The proposed pipeline delivers accurate results for synthetic spherical lesions and brain ROIs, with and without background activity consideration, enabling the evaluation of new attenuation correction approaches without utilizing measured PET emission data. Additionally, it offers a consistent method to generate realistic lesion ROIs, potentially applicable in assessing further PET correction techniques.

## Introduction

Positron emission tomography (PET) attenuation correction (AC) is crucial for accurate PET tracer's quantification [1]. Hybrid PET Magnetic Resonance Imaging (PET/MRI) gained interest due to its high soft-tissue contrast resolution, especially for neurological [2] oncology applications [3] and its low exposure to ionizing radiation compared to hybrid PET Computed Tomography (PET/CT), especially for pediatric patients. Another distinct strength of PET/MRI is cardiovascular/cardiac imaging when performed simultaneously. Although hybrid PET/MRI imaging systems were introduced a decade ago, they are still mainly used in the research arena. This is due to their cost and the lack of unique applications necessitating simultaneous PET and MR acquisition. In addition, PET AC using MRI information is not straightforward. MRI images provide information about tissue proton density and relaxation times but not tissue electron densities. Thus, body tissues with low proton densities or with very short relaxation time are not seen by the MRI system. The most important consequence is the absence of bone tissues in MRI based AC (MRAC) images affects the quantitative accuracy of the PET reconstructed images [4]. Research literature about MRAC for PET is generally categorized into: (1) MRI image tissue segmentation, like DIXON and UTE for identifying various tissues [5]; (2) templates-atlas-based approaches, such as superimposing a bone template on a Dixon attenuation map [6]; (3) deep learning techniques [7]; and (4) approaches based on PET emission data, e.g., MLAA reconstruction methods [8]. While MRAC issues have been resolved for healthy subjects, particularly in brain studies [9]; challenges persist for subjects with skull and brain abnormalities due to dataset limitations for template-atlas and deep learning MRAC approaches, requiring further evaluation and improvement in diverse patient cohorts [10].

Virtual synthetic PET imaging, utilized to evaluate new PET image reconstruction and analysis algorithms, can be categorized into three primary techniques.First, Monte Carlo simulations (MCS), which leverage random sampling to simulate ionizing radiation interaction with matter, have been validated and widely utilized to simulate realistic PET data across varied patient anatomies and pathologies [11–13]. Second, deep learning-based image generation, due to recent GPU software and hardware advances, has gained notable attention [14]. Lastly, the third category encompasses analytical-based image generation techniques, with various toolkits and approaches proposed in existing literature [15–17]. Our team developed a PET synthetic lesion insertion tool for the Siemens mMR PET/MRI scanner, validated its accuracy with recovery coefficients in the NEMA IEC phantom, and assessed MRAC to CTAC lesion activity bias in brain and pelvis regions [18]. Despite accurate results and simulations under 5 min, the tool lacked automated, consistent ROI insertion, especially in brain uptake studies. Extending our work, we devised an automatic pipeline, merging the Siemens mMR-specific tool with FreeSurfer software, to generate synthetic PET data for neurological studies and assess the PET quantitative accuracy of existing MRAC attenuation correction approaches.

## Method

The Siemens Biograph mMR PET/MRI system, utilized in this study, integrates simultaneous PET and MRI subsystems with 50 cm field of view (FOV) operating in 2D and 3D modes. Detector assembly for the PET sub-system part consist of 56 detector cassettes, each housing eight axially distributed 8 × 8 lutetium oxyorthosilicate crystal arrays linked to a 3 × 3 APD array for scintillation light readout. Complete camera description and geometry details are available in [19].

### Patient data

This study utilized neuro PET/MR datasets from eleven patients (median [IQR] age: 70 [65, 72.5] years old, 7 Females) acquired at the Washington University Knight Alzheimer Disease Research Center (ADRC), with approval from the institutional review board and patient consent. Tri-modality brain PET/MRI/CT images were acquired using Biograph mMR PET/MRI and Biograph True Point 40 PET/CT systems (Siemens Healthcare). Head CT images were obtained with the clinical Biograph True Point 40 PET/CT system. The quantitative accuracy of the brain PET images was evaluated using four MRAC approaches and compared to CTAC as a reference. The two-point DIXON MRI sequence (DIXON) [5] segmenting head tissue as fat and water like only, the same two-point DIXON but including a skull model (DIXONbone) [20], the ultra-short echo-time MRI sequence (UTE) [21] that extract bone information from short relaxation time of protons in bone, and a DIXON-trained deep-learning-network-generated pseudo-CT map [22]. Three MRAC approaches, DIXON, DIXONbone, and UTE, are available on the mMR PET/MRI system. T1 Magnetization-prepared Rapid Acquisition Gradient Echo (MPRAGE) MRI images were processed with FreeSurfer to provide a patient-specific brain atlas of 16 regions, which were used to define lesion shape and location in the synthetic lesion insertion tool.

### PET imaging

Patients were injected with an F-18-based amyloid-binding radio-ligand (Florbetapir). Data were acquired 50 min post-injection for 20 min from 10 patients, and immediately after injection for 70 min from 1 patient, using the Biograph mMR PET/MRI system. List-mode files were acquired and re-binned to sinograms using the Siemens research reconstruction software e7tools (Siemens healthcare). PET images were reconstructed using a 3D OSEM algorithm at 3 iterations, 24 subsets, and a 4 mm post-reconstruction Gaussian smoothing kernel [23]. The PET reconstructed image sizes are 344 × 344 × 127 voxels at 2.08 × 2.08 × 2.03 mm^3^ each.

### CT imaging

Low-dose brain CT images were acquired using the CT subsystem of the Biograph TruePoint 40 PET/CT scanner at 120 kVp, 25 mAs exposure. Images were reconstructed using the filtered back-projection algorithm with H19f. The dimensions of the brain CT images are 512 × 512 × 70 voxels at 0.59 × 0.59 × 2 mm^3^ per voxel.

### MRI imaging

Three brain MRI images were acquired using the Biograph mMR PET/MRI system using vendor-provided sequences, the standard two-point Dixon–volumetric interpolated breath-hold examination (VIBE), the high-resolution two-point Dixon CAPI, UTE, and the MPRAGE.

MRI T1-weighted brain images were acquired using a 3D MPRAGE sequence with the following imaging parameters: TE/TR = 2.95/2300 ms, TI = 900 ms, number of partitions = 176, matrix size = 240 × 256 × 176, voxel size = 1.05 × 1.05 × 1.2 mm^3^, acquisition time = 5 min 11 s. The T1-weighted image was used as an input to FreeSurfer to generate the patient specific brain atlas.

### Attenuation maps

#### DIXON

The DIXON attenuation map was acquired using a vendor-provided two-point Dixon VIBE MRI sequence with a 10° flip angle (FA). At repetition time (TR), 3.6 ms, there are two echo-time TE readouts, in-phase, 2.46 ms, and out-phase, 1.23 ms, from which fat and water dominant images are generated. The acquisition time was 19 s. Four classes of tissues are generated for whole-body PET/MRI applications: air, fat, and soft tissues, to which fixed 511 keV photons attenuation coefficient were assigned. In this study, the whole brain and the skull are considered uniform soft tissues (water). The dimensions of the DIXON images are 192 × 126 × 128 voxels, and the voxel size is 2.6 × 2.6 × 3.12 mm^3^.

#### DIXONbone

The DIXONbone images were generated based on the high-resolution DIXON Controlled Aliasing in Parallel Imaging Results in Higher Acceleration (CAIPIRINHA) images (TE1/TE2/TR = 1.28/2.51/4.14 ms, FA = 10°, dimensions = 384 × 204 × 132 voxels, and voxel size = 1.30 × 1.30 × 2.02 mm, acquisition time = 39 s). Skull bones linear attenuation coefficient (LAC) replaced the soft tissues LAC in the high-resolution DIXON images. The skull bones were generated using a vendor-provided model-based bone prototype segmentation algorithm (Siemens AG, Erlangen, Germany). The first step is to generate a bone model from pre-aligned MRI images and bone masks containing continuous bone LAC at 511 keV photons. In the second step, the patient DIXON image is registered to the generated MRI model, then bone masks are registered to the patient DIXON image, segmented in the bone tissues, and brought back to the original DIXOM image space using the same transformations [20, 24].

#### UTE

The UTE images were generated using a vendor-provided MRI imaging sequence with a 10-degree FA, 4.64 ms TR, and 0.07 ms and 2.46 ms TE, which results in simultaneous generation of cranial bones and the brain tissues. The acquisition time was 144 s. The resulted images were segmented into two compartments, soft tissues for the whole brain and bones-tissues for the cranial bones. The size of the raw UTE images consists of 192 × 192 × 192 voxels, 1.56 × 1.56 × 1.56 mm3 per voxel.

#### DL-DIXON

Synthetic pseudoCT attenuation maps were generated using a deep-learning technique. A network that combines the 3D residual and UNet architectures (ResUNet) was used. Pseudo-CT images were generated from the standard in- and opposite-phase DIXON images. More details about the DL-DIXON attenuation maps generation methodology, network architecture, training, and testing datasets were published in previous work in [22].

### Attenuation maps preprocessing

The four MRI-derived attenuation maps and CT images obtained directly from the Biograph mMR, and True Point 40 PET/CT systems, respectively, were resampled using nearest neighbor interpolation onto the default attenuation map gridded on 344 × 344 × 127 voxels at 2.086 × 2.086 × 2.031 mm^3^/voxel. The DIXON, DIXONbone, and DL-DIXON MRAC were registered to the UTE attenuation map using a 12-parameter affine registration with the FMRIB Linear Image Registration Tool (FLIRT) in the FSL toolbox [25].

The CT and pseudo-CT Hounsfield (HU) unit were converted to 511 keV linear attenuation coefficients by piecewise linear scaling [26]. The CT attenuation maps were aligned to DIXON, DIXONbone, UTE, and DL-DIXON attenuation maps using a 12-parameter affine registration with FLIRT. All MR and CT attenuation maps were then wrapped to default attenuation map space using vendor-provided e7tools software.

### Pipeline description

**Fig. 1:**
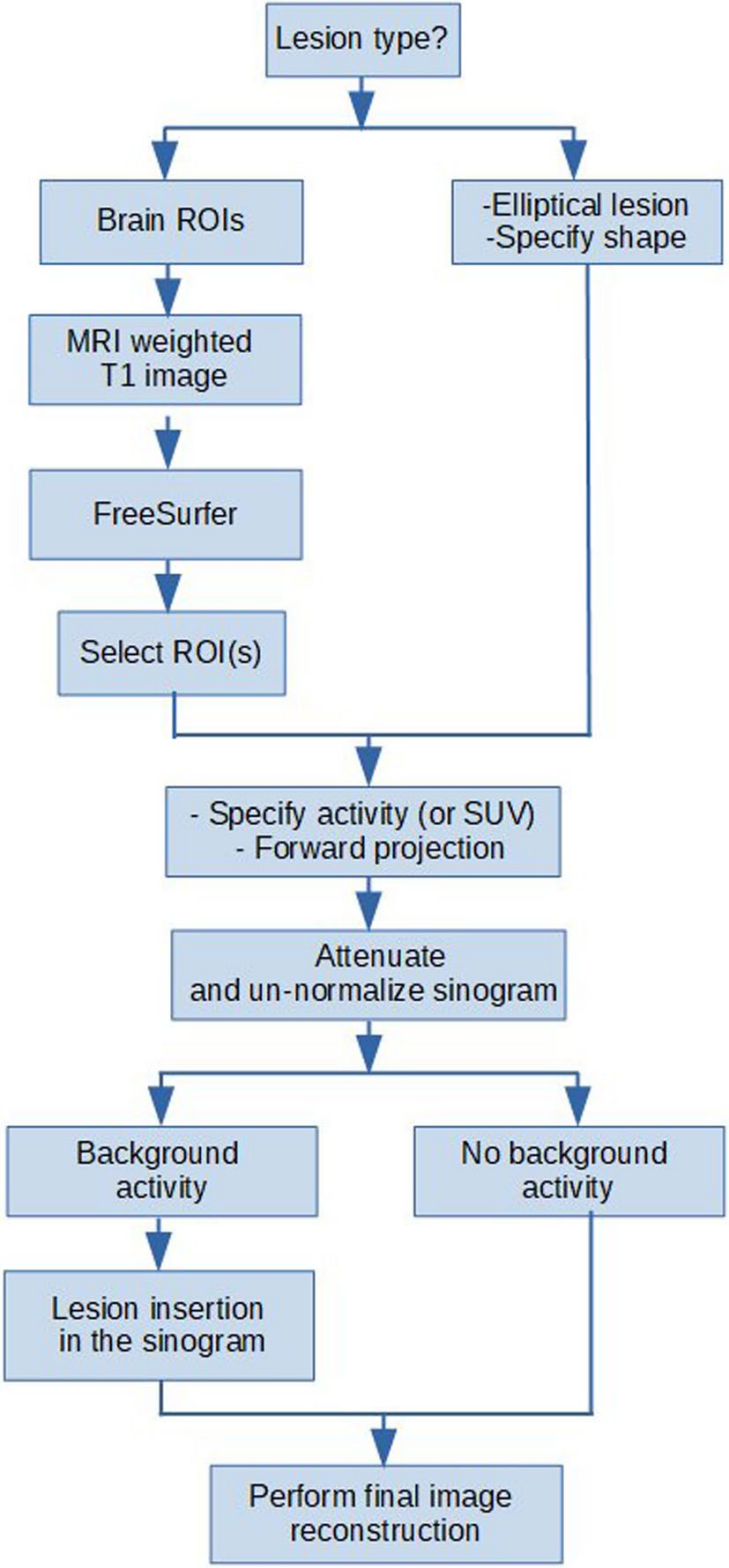
Pipeline for the evaluation of different PET attenuation correction approaches using the synthetic lesion ROIs insertion tool and FreeSurfer

Our pipeline, visualized in Fig. 1, combines a validated synthetic lesion insertion tool for Siemens mMR [18] and FreeSurfer framework for brain segmentation, utilizing T1-weighted MRI images to produce a patient-specific brain atlas. Brain regions of interest (ROIs), with definable activity or Standardized Uptake Value (SUV), are input into the tool, optionally using original PET image data for lesion-to-background ratio (LBR) application and smoothing via the scanner's point spread function (PSF). Subsequent to this, lesion ROI and attenuation map images are forward-projected, with the lesion ROI sinogram undergoing multiple processing stages—including voxel-wise division by extended normalization and integral factors matrices, calibration of lesions activity, calculation and addition of scatter using e7tools, and Poisson noise addition—before being added to or replacing sinogram counts in the patient PET projection space. Final image reconstruction is achieved using standard 3D-OSEM with three iterations and 21 subsets [23]. More technical details about the lesion insertion tool are presented in [18].

### Brain regions of interest

**Fig. 2:**
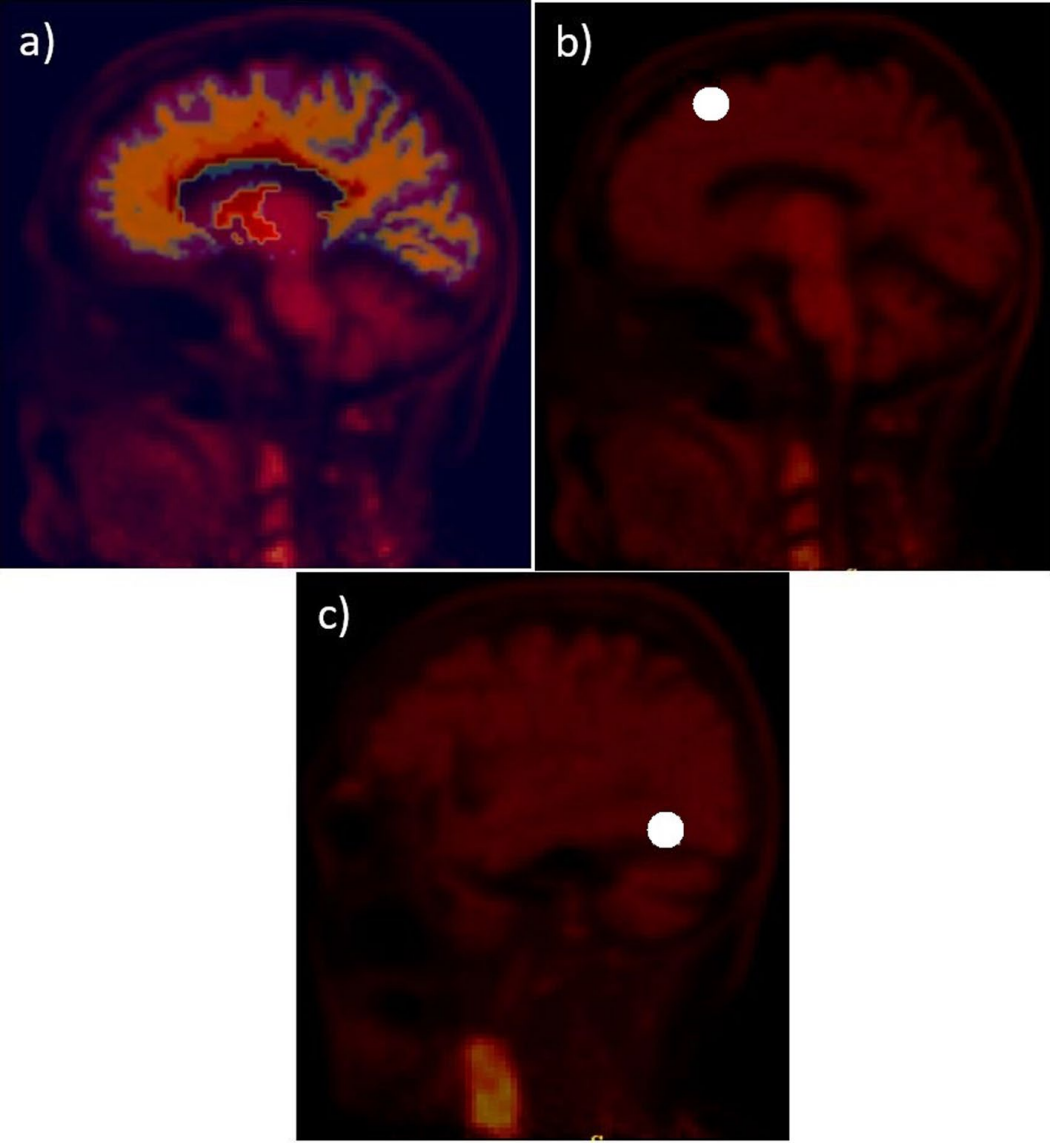
Examples of FreeSurfer brain atlas (**a**) and two spherical lesions (**b** and **c**). The atlas and the inserted lesions are superimposed over the PET image

In a typical comparison of PET/MRI to PET/CT for attenuation correction evaluation, PET emission data were reconstructed with two different attenuation maps: a specific MRAC and a CTAC. For regional brain uptake analysis, brain ROIs are delineated using manual or automatic approaches to calculate the uptake deviation from MRAC PET reconstruction relative to CTAC PET reconstruction. In the case of automatic ROIs generation, for instance, a brain atlas generated from a FreeSurfer T1 weighted MRI images with 256 × 256 × 256 voxels at ~ 1 mm^3^/voxel needs to be in the same space as the final reconstructed PET images. The FreeSurfer brain atlases were aligned to the PET using rigid registration with FSL's FLIRT. Figure 2a presents a 2D slice of a brain atlas superimposed on its corresponding 2D brain PET image. Brain ROIs are defined, projected to sinogram space, and reconstructed with and without considering the patient's sinogram, using a lesion insertion tool. The MRAC to CTAC bias is then compared in the inserted 16 brain ROIs and the original PET images.

### Pipeline evaluation

Two spherical 4 mm radius lesions were inserted, one in the superior frontal cortex and another in the fusiform gyrus. Figure 2b, c show examples of the two spherical lesions inserted in the brain. The MRAC to CTAC bias is most sensitive to lesion location; a lesion inserted near the skull shows a higher bias than one inserted farther from the brain skull. PET images with inserted lesions were reconstructed with and without the brain PET background (or projections) using the four MRAC maps, DIXON, DIXONbone, UTE, DL-DIXON, and the CTAC map. In addition, PET images without the inserted lesions were also reconstructed using the same MRAC maps. PET reconstruction bias in the inserted lesions ROIs were compared across methods.

#### Data analysis

MRAC to CTAC bias was calculated in the inserted spherical lesions ROIs and FreeSurfer brain atlas ROIs using relative and relative absolute errors in Eq. 1 and Eq. 2, respectively. Box plots of the relative and absolute MRAC to CTAC bias were displayed for the entire patient cohort.

1 $$Relative \;bias = \left( {PET_{MRAC} - PET_{CTAC} } \right)/PET_{CTAC}$$

2 $$Aboslute\; bias = \left| {\left( {PET_{MRAC} - PET_{CTAC} } \right)} \right|/PET_{CTAC}$$

#### Statistical analyses

Statistical analyses were performed using R 4.2.0 (Foundation for Statistical Computing, Vienna, Austria). Comparisons between PET absolute bias using DL-DIXON AC maps and using other three MRAC maps were performed using paired t tests with the Benjamini–Hochberg to control for the false discovery rate in multiple comparisons.

## Results

### Pipeline evaluation

**Fig. 3:**
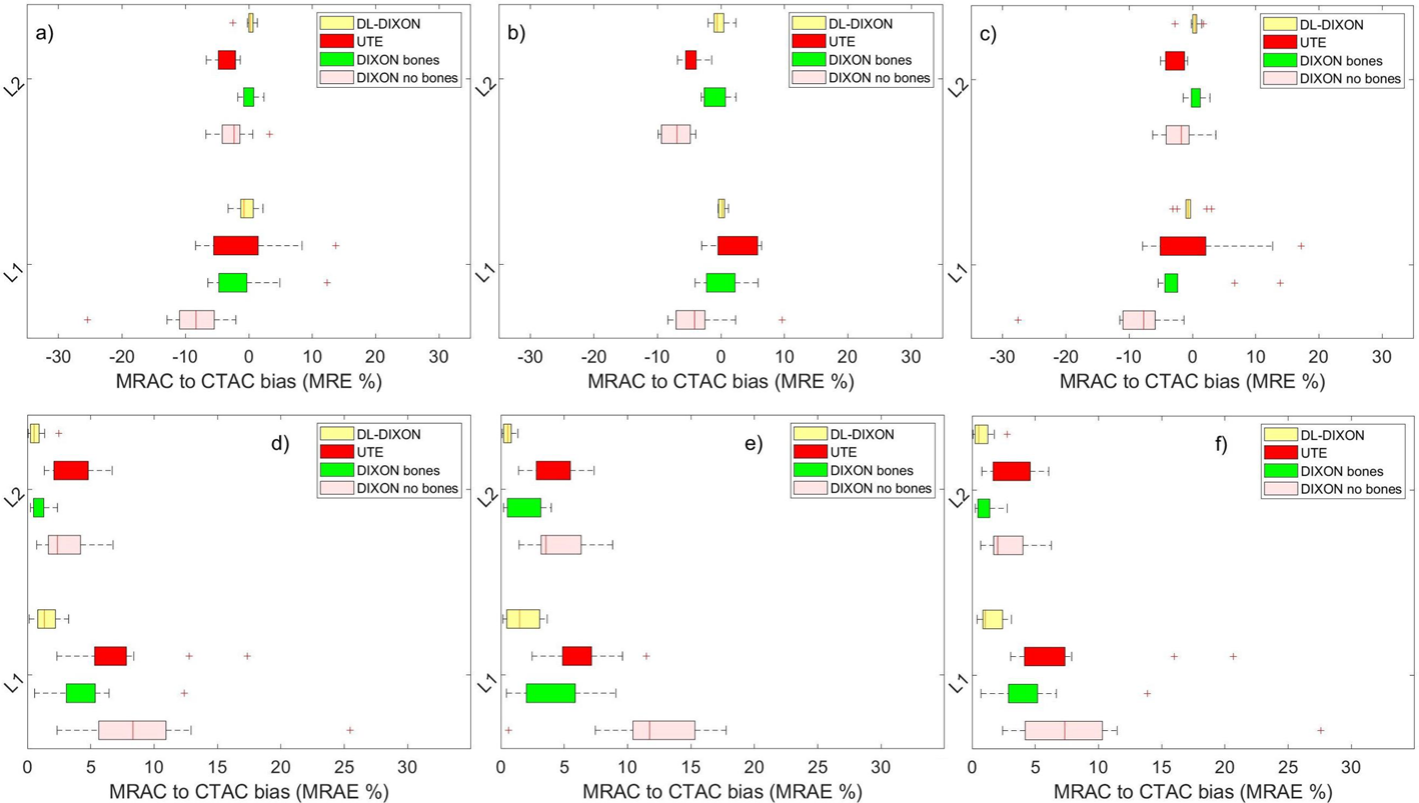
MR to CT-based PET attenuation correction relative (**a**–**c**) and absolute (**d**–**f**) bias in two synthetic spherical lesions inserted at two brain locations. Lesions were reconstructed with (**a** and **d**) and without (**b** and **e**) the background activity and from the corresponding spherical ROIs in the original PET images (**c** and **f**)

Figure 3 presents a boxplot of the MRAC to CTAC lesion's activity bias for the 11 patient datasets. Figure 3a–c show MRAC to CTAC bias in lesions reconstructed with and without background activity and in the original PET images, respectively. The original PET images show similar behavior to the inserted lesions for the four MRAC approaches. For lesions inserted in the superior frontal cortex, at the vicinity of the skull, the DIXON AC showed the largest underestimation of activity with a median and an interquartile range (IQR) of − 8.33% [− 10.93%, − 5.45%] for lesion inserted in the activity background. Lesion inserted without the activity background showed a median [IQR] of − 11.74 [− 15.32%, − 10.42%]. The UTE showed an improved activity estimation compared to the DIXON AC, with a median [IQR] of − 5.26% [− 4.73%, − 0.30%] for lesion inserted with the background activity and a median [IQR] of − 4.14% [− 5.86%, − 0.84%] for lesion inserted without the background activity. The DIXONbone showed similar performances to the UTE with a median [IQR] of − 2.32% [− 4.19%, − 1.4%] in lesions inserted with the background activity, and a median [IQR] of − 3.56% [− 6.33%, − 3.18%] in lesions inserted without the background activity. The DL-DIXON showed the best performance, with a median [IQR] bias of − 0.03% [− 0.80%, 0.78%] for lesions inserted in the background activity and a median [IQR] bias of − 0.88% [− 3.16%, 0.03%] for lesions inserted without the background activity.

In lesion inserted in the fusiform gyrus, not in the vicinity of the skull, the four attenuation maps showed lower MRAC to CTAC activity bias than those from the lesion inserted in the superior frontal cortex. The DL-DIXON has the least bias, followed by DIXONbone, UTE, and DIXON. The median [IQR] bias were − 5.26% [− 5.54%, 1.50%] and − 0.75% [− 1.26%, 0.72%] from DIXON and UTE for lesions inserted with the background activity. Lesion inserted without the background showed median [IQR] bias of − 6.03% [− 7.16%, − 4.87%] and median [IQR] bias of 3.06% [− 3.07%, − 0.01%] for lesions inserted without considering the background. The DL-DIXON showed a consistent lesion activity bias to lesion inserted in the prefrontal cortex with a median [IQR] of 0.24% [0.01%, 0.67%] for lesions inserted on the background the medium [IQR] of − 0.23% [− 0.20, 0.73%] on lesion inserted without the background.

For lesions inserted in the background activity (Fig. 3d), DL-DIXON showed a significantly smaller PET absolute bias than DIXON (*p* < 0.01) and UTE (*p* < 0.01) in both inserted lesions. DL-DIXON also showed a significantly smaller PET absolute bias than DIXONbone (*p* < 0.05) in the lesion inserted in the superior frontal cortex. For lesions inserted without background activity (Fig. 3e), DL-DIXON showed a significantly smaller PET absolute bias than DIXON (*p* < 0.001), DIXONbone (*p* < 0.05), and UTE (*p* < 0.001) in both inserted lesions. For the original brain PET reconstructed images (Fig. 3f), DL-DIXON showed a significantly smaller PET absolute bias than DIXON (*p* < 0.01) and UTE (*p* < 0.01) in both inserted lesion regions. DL-DIXON also showed a significantly smaller PET absolute bias than DIXONbone (*p* < 0.01) in the inserted lesion region in the superior frontal cortex.

### Regional brain ROIs

**Fig. 4:**
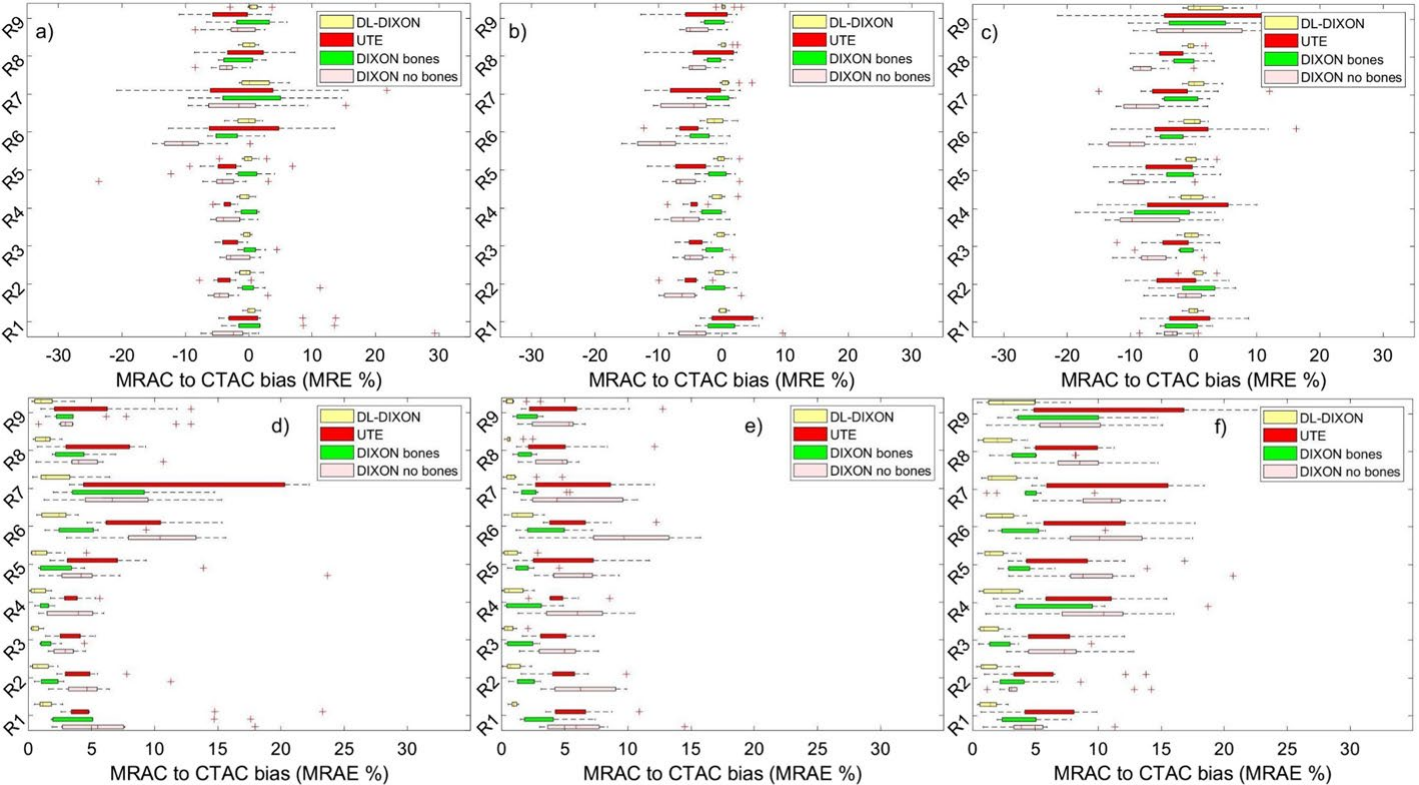
MR to CT based PET attenuation correction relative (**a**–**c**) and absolute (**d**–**e**) bias in FreeSurfer ROIs in the prefrontal cortex. ROIs were reconstructed with (**a** and **d**) and without (**b** and **e**) background activity and from the original PET images (**c** and **f**)

Figure 4 presents MRAC to CTAC activity bias in nine lesion ROIs in the prefrontal cortex. MRAC to CTAC activity bias in lesion ROIs inserted with and without the background activity is presented in Fig. 4a–c shows activity bias in the original reconstructed PET images using the same ROIs.

Lesion ROIs inserted in the prefrontal cortex with and without the background activity showed similar behavior to lesion ROIs in the original brain PET reconstructed images. However, a slightly higher fluctuation, activity bias range, was observed in the original lesion ROIs due to their lower statistics than the inserted synthetic lesion ROIs. For lesion ROIs in the prefrontal cortex, inserted on the background activity, the DIXON AC showed the largest underestimation of activity, with a median ranging from − 1.75% in the left frontal pole to − 10.43% in the caudal middle frontal gyrus and present the lowest and highest MRAC to CTAC activity biases, respectively (Fig. 4). The UTE AC showed an enhancement in activity estimation relative to the DIXON, with an MRAC to CTAC activity bias with a median ranging from − 0.22 to − 4.17% for both lesions inserted with or without the background activity. However, UTE has higher interpatient variability, IQR (Fig. 4a). The DIXONbone performed better than the UTE, with a median activity deviation ranging from − 2.77 to 1.91%. The DIXON-DL presented the best performance across all MRAC approaches, with the lowest MRAC to CTAC activity bias with a medium ranging from − 0.39 to 0.4%. A similar pattern was observed using MRAC to CTAC activity bias in lesion ROIs inserted without considering the background activity and lesion ROIs in the original reconstructed images (Fig. 4b). MRAC to CTAC lesion ROIs bias shows good agreement with MRAC to CTAC bias calculated with the same ROIs in the original brain PET images. However, the latter have more interpatient variability (Fig. 4c).

**Fig. 5:**
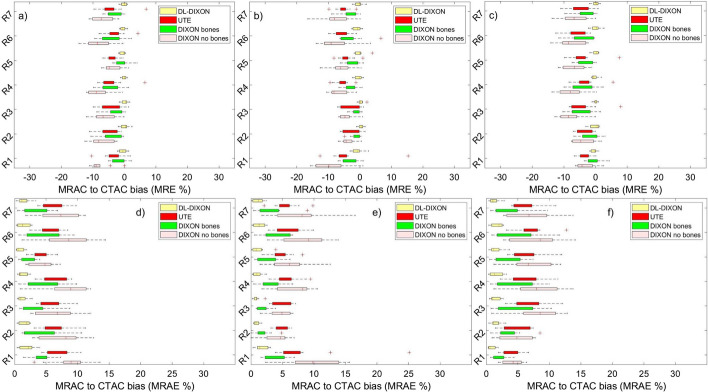
MR to CT based PET attenuation correction bias in a FreeSurfer extracted T1 ROIs in the parietal and temporal cortex. For relative (**a**–**c**) and absolute bias (**d**–**e**), ROIs were reconstructed with and without background activity (**a**, **b**) and the original PET images in (**c**). Same order is followed in Sub-figures (**d**–**f**)

Figure 5 depicts MRAC to CTAC bias in brain ROIs in the parietal and temporal cortexes. Lesion ROIs inserted with and without the background activity show similar MRAC to CTAC bias for the four MRAC approaches. The median MRAC to CTAC activity bias of the 16 inserted lesion ROIs for the 11 patients with or without the background activity and in the original PET images followed the same pattern.

For lesion ROIs inserted in the background activity (Figs. 4d and 5d), DL-DIXON showed a significantly smaller PET absolute bias than DIXON (p < 0.05) and UTE (*p* < 0.05) in all 16 ROIs. Except in medial orbital gyrus, the orbital part of inferior frontal gyrus and the triangular part of inferior frontal gyrus, DL-DIXON also showed a significantly smaller PET absolute bias than DIXONbone (*p* < 0.05). For lesion ROIs inserted without background activity (Figs. 4e and 5e), DL-DIXON showed a significantly smaller PET absolute bias than DIXON (*p* < 0.01) and UTE (*p* < 0.05) in all 16 ROIs. Except in the orbital part of inferior frontal gyrus and the anterior part of middle frontal gyrus, DL-DIXON showed a significantly smaller PET absolute bias than DIXONbone (*p* < 0.05). For the original brain PET reconstructed images (Figs. 4f and 5f), DL-DIXON showed a significantly smaller PET absolute bias than DIXON (*p* < 0.05) and UTE (*p* < 0.01) in all 16 ROIs. Except in the opercular part of inferior frontal gyrus, DL-DIXON also showed a significantly smaller PET absolute bias than DIXONbone (*p* < 0.05).

## Discussion

Our developed pipeline analytically generates realistic 3D PET brain ROIs and lesions. The tool was used to evaluate the quantitative accuracy of different MRI-based PET attenuation correction approaches and demonstrated a very good agreement with MRAC to CTAC assessed in the original measured patient PET data and will enable mimicking clinically relevant diseases in the brain studied by PET. Different brain anatomical regions, including the prefrontal, parietal, temporal cortexes, and the fusiform gyrus, were investigated. The study presents three sets of result, lesions with and without PET patient emission data and original PET images. Lesions inserted without PET emission data exhibited similar behavior to those inserted with it (emission data) and in the original reconstructed PET images. Findings align with existing literature [6] and advocate for using the synthetic lesion insertion tool to evaluate quantitative accuracy without requiring PET emission data.

Our analytical synthetic lesion insertion approach has practical advantages over the widely used MCS toolkits like GATE in PET imaging [27]. It significantly reduces simulation time, from days on a CPU cluster to 15 min on a standard workstation (with an Intel(R) Xeon(R) CPU X5650 at 2.67 GHz, housing 2 processors). Moreover, it offers an easy integration into existing PET imaging pipelines or workflows, utilizing a scanner geometry-specific forward projector. Unlike the GATE approach, which requires extensive validation for generating realistic PET datasets, our method for synthetic PET data creation is considerably straightforward.

Analytical lesion insertion tools, such as PETSTEP introduced by Beatrice et al. [15] are designed to enhance PET imaging evaluations, particularly in automatic segmentation. PETSTEP, which inserts lesions into a previously reconstructed PET image's projection space using MATLAB’s Radon transform, ensures independence from scanner geometry. However, it mandates that lesions be at least 3 cm from the phantom edge, necessitates specific scanner calibration, and is not ideal for assessing PET/MRI image quantitative accuracy near structures like cranial bones. Pfaehler et al. [16] introduced another lesion insertion tool called SMART, which utilizes the real attenuation map instead of water and offers 3D lesion insertion capabilities. Nevertheless, SMART requires calibration for the scanner in use and hasn’t been validated with patient data. Another methodology by C Tsoumpas et al. [17], used the STIR reconstruction software [28] and segmented 4D anatomical MRI images to generate 4D PET data for evaluating motion correction algorithms. Yet, STIR remains time-consuming, taking several hours for forward projection and PET image reconstruction. The proposed automatic synthetic PET generation combine the advantage of generating realistic lesion and ROIs for specific scanner, the Siemens mMR, while offering reduced simulation time.

In our study, lesion ROIs are attenuated utilizing the real attenuation map, which limits quantitative bias compared to other studies that assume water for lesion ROIs attenuation. Nevertheless, this approach necessitates the precise registration of the brain atlas to the PET, and ensuring no apparent interference with the skull which can lead to a bias in the lesion ROIs activity. While the current noise simulation is implemented for static PET images, limiting the capabilities for simulating dynamic PET data, however it can be readily adapted for dynamic PET scenarios where noise need to be extracted from each sinogram frame and added into the lesion ROIs. Additionally, both the forward projector and the reconstruction tool are provided by the manufacturer, necessitating research collaboration between labs and the vendor. An analysis of the original PET images indicated that larger ROIs display more significant fluctuations in MRAC to CTAC bias than smaller ones. This variation might stem from the reduced statistical data in the original lesion ROI images, in contrast to the synthetically inserted lesions.

The proposed automatic pipeline accelerates the development and assessment of PET/MRI attenuation correction approaches and goes beyond this application. The availability of ground truth data enables broader evaluation of PET data correction algorithms, including scatter, motion, and partial volume effect corrections, and enhances PET image analysis, such as segmentation and disease classification. Additionally, the pipeline facilitates the synthesis of PET images exhibiting unusual uptake patterns, thereby establishing a reliable ground truth that is vital for the rigorous evaluation and training processes associated with deep learning algorithms.

## Conclusion

A pipeline based on a previously developed and validated lesion insertion tool and FreeSurfer framework was proposed to accelerate the development, evaluation, and transition of different PET/MRI attenuation correction approaches to clinical neurological applications. Four MRI-based PET attenuation correction approaches were evaluated against the CT attenuation map. Three types of evaluation were presented, MRAC to CTAC in lesions inserted with the background, without considering the background, and lesion ROIs in the original PET reconstructed images. MRAC to CTAC in inserted lesions, with and without background, is consistent with MRAC to CTAC bias in the original reconstructed PET images. This led us to conclude that the background activity does not show an apparent effect on MRAC to CTAC bias behavior. Thus, the proposed pipeline enables evaluation of novel MRI-based PET attenuation correction methods without requiring actual patient PET emission data. Furthermore, this pipeline offers a consistent and straightforward means to simulate authentic PET data, which can then be employed in assessing PET correction techniques such as scatter and motion correction, as well as in deep learning applications. The lesion insertion tool will be available for online users and can be used for multiple purposes.

## Data Availability

The datasets used and/or analyzed during the current study are available from the corresponding author on reasonable request.
